# Gender-specific associations between serum docosahexaenoic acid levels and chronic pain prevalence: a cross-sectional study

**DOI:** 10.3389/fmed.2025.1563209

**Published:** 2025-05-27

**Authors:** Li Zhang, Hai Xu, Lihua Hang, Xin Lin

**Affiliations:** ^1^Department of Anesthesiology, The First People’s Hospital of Kunshan Affiliated with Jiangsu University, Kunshan, China; ^2^Kunshan Biomedical Big Data Innovation Application Laboratory, Jiangsu, China; ^3^Kunshan Cancer Pain Prevention and Treatment Key Laboratory, Jiangsu, China

**Keywords:** chronic pain, polyunsaturated fatty acids, serum docosahexaenoic acid, cross-sectional study, NHANES

## Abstract

**Background:**

This study examines the relationship between serum docosahexaenoic acid (DHA) levels and chronic pain prevalence, with emphasis on gender differences and smoking status interactions.

**Methods:**

Data from 1,677 adults participating in the 2003-2004 National Health and Nutrition Examination Survey (NHANES) were analyzed. Chronic pain was defined as pain persisting ≥ 3 months. Relationships between serum DHA levels and chronic pain were assessed using logistic regression and generalized additive models, adjusting for relevant covariates.

**Results:**

Among participants (median age 46 years), 17.9% reported chronic pain. In females, a non-linear L-shaped association was observed: for DHA levels < 187 μmol/L, each 10-unit increase was associated with lower chronic pain prevalence (adjusted OR: 0.92; 95% CI: 0.87-0.97; *P* = 0.0019), while at levels ≥ 187 μmol/L, this protective association diminished (adjusted OR: 1.03; 95% CI: 0.99-1.08; *P* = 0.1578). In males, no overall significant association was found (adjusted OR: 1.0; 95% CI: 0.97-1.03; *P* = 0.9057); however, a significant interaction with smoking status was detected (P-interaction = 0.0062). Among non-smoking males, higher DHA levels were associated with increased chronic pain odds (OR: 1.06; 95% CI: 1.01-1.11; *P* = 0.0220), while no significant association was observed among male smokers.

**Conclusion:**

The relationship between serum DHA and chronic pain demonstrates gender-specific patterns, with a threshold effect in females and smoking-dependent associations in males. These findings suggest that the relationship between DHA and chronic pain involves complex biological interactions that vary by gender and smoking status, highlighting the need for personalized approaches in future research and interventions.

## Introduction

Chronic pain, termed as pain remaining for no less than 3 months, is a common and debilitating issue impacting millions of people globally. It greatly diminishes quality of life and places a considerable strain on healthcare systems (1). Although substantial research has been conducted, the underlying mechanisms of enduring pain are still incompletely comprehended, and effective treatment options are scarce. Recent investigations indicate that dietary components, especially polyunsaturated fatty acids (PUFAs), could influence pain perception and inflammation (2–4). Among these, docosahexaenoic acid (DHA), a form of polyunsaturated fat, has attracted interest due to its possible anti-inflammatory and neuroprotective effects (3, 5–7). DHA acts as an essential component in nerve cell membranes and contributes to reducing inflammation (2). Research-based studies have demonstrated that DHA can modulate pain pathways and reduce inflammatory responses (8). A Mendelian randomization study found an inverse relationship between DHA concentrations and the presence of abdominal, pelvic, and lower back discomfort (9). Epidemiological studies also indicate an opposite connections between the consumption of omega-3 fatty acids and the incidence of long-term pain disorders (10). Nevertheless, a 5-year study with random assignment and comparison groups performed in the American reported no improvement in osteoarthritis-related knee pain with omega-3 FA supplementation (11). Thus, the cause-related connection between PUFAs and persistent discomfort persists. Additionally, the precise connection between serum DHA levels and chronic pain has not been comprehensively explored in large-scale community research.

Utilizing the comprehensive data collection and representative sampling of the U.S. population offered by NHANES, this research provides an important foundation for exploring the relationship in question. This study draws on data from the 2003-2004 NHANES cycle to evaluate the relationship between serum DHA concentrations and persistent pain in grown individuals. This study aims to deliver strong evidence regarding this potential connection by employing sophisticated statistical techniques, such as generalized additive models (GAM) to analyze non-linear relationships.

A significant advancement of this study is the examination of gender differences and the potential relationship between smoking status and serum DHA levels in the context of chronic pain prevalence. Our investigation explores whether serum DHA concentrations are associated with variations in chronic pain prevalence across different population groups. This cross-sectional analysis cannot establish causality, but it may generate hypotheses for future longitudinal studies. Through a thorough examination of NHANES data and statistical adjustment for possible confounding variables—including demographic factors, comorbidities, and lifestyle habits—this research aims to provide insights into the complex relationship between serum DHA and chronic pain patterns observed in different population subgroups.

## Materials and methods

### Study population

NHANES is a health program aimed at evaluating the wellbeing and dietary status of the American people (12). This work employed data from the 2003-2004 cycle, involving 10,122 participants, to investigate the relationship among serum DHA concentrations and persistent pain.

**FIGURE 1:**
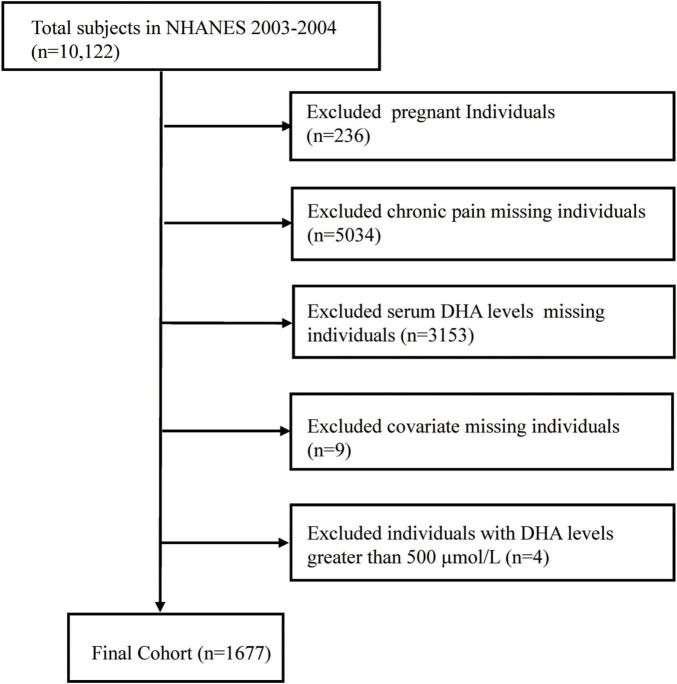
Research workflow diagram.

To ensure the analysis’s reliability and validity, a thorough participant selection process was implemented. Initially, pregnant individuals (*n* = 236) were removed from the study due to potential confounding variables. Next, those with incomplete data, including information on chronic pain (*n* = 5,043) and serum DHA levels (*n* = 3,153), were excluded from the study sample. Additionally, participants who did not have data on diabetes and smoking covariates, as well as those with missing C-reactive protein (CRP) values (*n* = 9), were also excluded, along with four outliers for DHA levels that exceeded 500 μmol/L. Ultimately, 1,677 participants were incorporated into the conclusive analysis, with the specific inclusion and exclusion criteria depicted in Figure 1. The NHANES research obtained authorization from the NCHS Ethical Oversight Committee, and every participant agreed to the terms by signing an informed consent document.

### Measurement of serum DHA and other fatty acids

Blood serum specimens were managed, stored, and dispatched to the Laboratory Sciences Branch of the National Center for Environmental Health at the Centers for Disease Control and Prevention for examination. Long-chain carboxylic acids were identified utilizing mass analysis of negative ions with electron capture, with results obtained in 34 min. Ten PUFAs were chosen for additional examination: α-linolenic acid (ALA, 18:3n-3), stearidonic acid (SDA, 18:4n-3), eicosapentaenoic acid (EPA, 20:5n-3), docosahexaenoic acid (DHA, 22:6n-3), linoleic acid (LA, 18:2n-6), γ-linolenic acid (GLA, 18:3n-6), eicosadienoic acid (EDA, 20:2n-6), dihomo-γ-linolenic acid (HGLA, 20:3n-6), arachidonic acid (AA, 20:4n-6), and docosapentaenoic acid (DPA, 22:5n-6).

### Assessment of chronic pain

Chronic pain was evaluated using a pain questionnaire administered during home interviews. All individuals aged 20 and older who took part in the survey were qualified. They were inquired whether they had suffered from pain lasting longer than 24 h during the previous month. If they answered yes, additional questions were asked regarding the length of their symptoms (under 1 month, from 1 month to under 3 months, from 3 months to under 12 months, or 12 months and above). As per the definition provided by the American College of Rheumatology (ACR), chronic discomfort in this research was characterized as pain persisting for 3 months or more. Participants who indicated no pain in the previous month or experienced pain lasting fewer than 3 months were grouped as the group without chronic pain.

### Other variables

Information on demographics, such as age, sex, racial/ethnic background, education attainment, and income-to-poverty ratio, was gathered. Participants were additionally inquired whether a healthcare provider had diagnosed them with chronic illnesses such as diabetes, as well as details regarding their smoking and alcohol consumption behaviors. The body mass index (BMI) was determined using height (in centimeters) and weight (in kilograms). CRP levels were also considered as additional variables.

### Statistical analysis

The research analysis was carried out utilizing EmpowerStats and R software. Baseline data were described according to tertiles of DHA concentrations. Differences in continuous variables across tertiles of DHA were assessed using survey-weighted medians and interquartile ranges (IQR), with *P*-values calculated using survey-weighted linear modeling. For discrete data, weighted survey proportions with 95% confidence intervals (CI) were computed, and the relationship with DHA tertiles was assessed using weighted chi-square tests, with *P*-values representing the significance of these relationships. Extreme values with DHA levels exceeding 500 μmol/L were excluded.

We employed logistic regression to evaluate the relationship between different factors and the occurrence of chronic pain, presenting findings as odds ratios (ORs) accompanied by 95% CIs. Confounding variables were chosen according to their significance to the results or if they changed effect estimates by over 10% (Supplementary Tables 1, 2) (13). Following the assessment of clinical relevance, the subsequent covariates were adjusted for: age, sex, BMI, ethnicity, tobacco use, educational background, poverty income ratio, diabetes status, and CRP.

A GAM was employed to explore the relationship between DHA concentrations and the prevalence of persistent discomfort, focusing on dose-response dynamics. To investigate the cutoff influence of serum DHA on persistent discomfort, we utilized a two-segment piecewise linear regression model. The critical juncture for DHA was determined through exploratory analysis, enhancing model likelihood. A likelihood ratio test based on log-likelihood values was employed to compare the linear model with the two-segment piecewise model.

We carried out a logistic regression analysis to explore how smoking status interacts with DHA exposure regarding the prevalence of persistent pain. A threshold of 0.05 or lower was used to assess notable statistical finding.

## Results

### The foundational attributes of the participants

The cohort included 1,677 individuals, aged between 20 and 85 years. The average age was 46 years, with 852 (50.82%) identified as female. The data were categorized into tertiles according to serum DHA concentrations. We noted a marked rise in age corresponding to ascending tertile groups, with the High DHA tertile displaying the oldest average age. Additionally, socioeconomic status, assessed through PIR, showed significant disparities among the tertile groups, where the High DHA tertile recorded the highest PIR value. Notable associations were identified between sex, ethnicity, educational attainment, and smoking habits with the tertile groups. In contrast, BMI, diabetes, and alcohol intake did not show significant relationships with the tertile groups. A summary of the participants’ baseline characteristics is provided in Table 1. In males, the chronic pain prevalence was 15.05% in the LowerDHA tertile (35.7–106 μmol/L), 12.59% in the Middle DHA tertile (107–153 μmol/L), and 13.22% in the High DHA tertile (154–489 μmol/L). Among females, the prevalence rates were 23.38, 18.35, and 17.13% for the respective tertiles (Table 2).

**TABLE 1:** General characteristics parameters according to the DHA in serum.

Variables	Total (*n* = 1,677)	Lower DHA (35.7–106 μmol/L) (*n* = 550)	Middle DHA (107–153 μmol/L) (*n* = 564)	High DHA (154–489 μmol/L) (*n* = 563)	*P*-value
Age, years	46.00 (34.00, 58.00)	40.00 (30.00, 51.00)	46.00 (34.00, 60.00)	52.00 (38.00, 65.00)	<0.0001
Gender, n (%)					<0.0001
Male	49.18 (47.47, 50.89)	54.99 (51.29, 58.63)	49.83 (45.71, 53.95)	41.61 (37.28, 46.08)	
Female	50.82 (49.11, 52.53)	45.01 (41.37, 48.71)	50.17 (46.05, 54.29)	58.39 (53.92, 62.72)	
Poverty income ratio	2.87 (1.61, 4.80)	2.69 (1.40, 4.45)	2.72 (1.64, 4.47)	3.28 (1.98, 5.00)	0.0051
BMI, kg/m^2^	27.48 (24.04, 31.21)	27.59 (23.91, 31.48)	27.65 (24.45, 32.04)	27.24 (23.84, 30.59)	0.1966
Race/ethnicity, n (%)					<0.0001
Mexican American	73.67 (64.82, 80.95)	77.65 (68.25, 84.89)	73.06 (64.26, 80.35)	69.55 (58.33, 78.85)	
Other Hispanic	10.52 (7.36, 14.84)	7.36 (4.71, 11.34)	13.15 (9.61, 17.74)	11.64 (6.90, 18.96)	
Non-Hispanic White	7.95 (4.42, 13.88)	10.01 (5.26, 18.21)	8.74 (4.44, 16.49)	4.71 (2.38, 9.09)	
Non-Hispanic black	4.83 (3.62, 6.42)	3.17 (1.48, 6.67)	2.13 (0.88, 5.07)	9.53 (6.86, 13.09)	
Other race	3.02 (1.41, 6.37)	1.80 (0.52, 6.06)	2.92 (1.19, 6.98)	4.58 (2.45, 8.40)	
Education, n (%)					0.0269
Less than high school	18.12 (14.78, 22.02)	17.45 (12.93, 23.14)	19.39 (14.41, 25.57)	17.64 (12.65, 24.07)	
High school diploma (including GED)	26.41 (23.94, 29.03)	31.45 (26.99, 36.28)	26.26 (21.71, 31.38)	20.56 (16.38, 25.49)	
More than high school	55.41 (51.46, 59.30)	51.02 (44.78, 57.23)	54.35 (47.72, 60.83)	61.70 (54.63, 68.31)	
Unknown	0.06 (0.01, 0.25)	0.07 (0.01, 0.57)	0.00 (0.00, 0.00)	0.09 (0.01, 0.78)	
Alcohol, n (%)					0.3203
No	73.16 (67.54, 78.12)	73.94 (65.74, 80.76)	74.62 (70.44, 78.40)	70.75 (63.78, 76.86)	
Yes	26.84 (21.88, 32.46)	26.06 (19.24, 34.26)	25.38 (21.60, 29.56)	29.25 (23.14, 36.22)	
Diabetes, n (%)					0.0627
No	92.85 (91.01, 94.34)	94.75 (92.19, 96.50)	92.94 (89.51, 95.31)	90.51 (86.12, 93.61)	
Yes	7.15 (5.66, 8.99)	5.25 (3.50, 7.81)	7.06 (4.69, 10.49)	9.49 (6.39, 13.88)	
Smoking status (self-reported), n (%)					<0.0001
Never smoking	24.49 (21.20, 28.11)	34.16 (30.53, 37.99)	23.94 (19.41, 29.14)	13.55 (9.23, 19.45)	
Former smoker	25.78 (22.33, 29.57)	20.83 (17.33, 24.83)	24.30 (20.65, 28.36)	33.16 (25.74, 41.51)	
Current smoking	49.73 (46.42, 53.04)	45.01 (40.05, 50.06)	51.77 (48.21, 55.31)	53.30 (46.47, 60.00)	
DHA, μmol/L	121.00 (91.20, 167.00)	82.50 (69.60, 95.80)	125.00 (115.00, 138.00)	195.00 (171.00, 236.00)	<0.0001
ALA, μmol/L	60.90 (46.10, 84.60)	48.80 (37.70, 66.00)	63.00 (50.20, 84.20)	76.80 (57.70, 101.00)	<0.0001
SDA, μmol/L	686.00 (591.00, 801.00)	615.00 (535.00, 731.00)	699.00 (617.00, 811.00)	755.00 (657.00, 882.00)	<0.0001
EPA, μmol/L	41.40 (29.60, 60.40)	30.70 (21.70, 39.60)	41.50 (31.20, 53.10)	65.40 (46.80, 96.50)	<0.0001
DPA, μmol/L	19.10 (15.20, 25.30)	17.20 (14.00, 20.80)	20.80 (16.50, 26.10)	21.10 (15.20, 29.30)	<0.0001
LA, μmol/L	3430.00 (2970.00, 3960.00)	3160.00 (2760.00, 3620.00)	3530.00 (3120.00, 3990.00)	3690.00 (3240.00, 4190.00)	<0.0001
GLA, μmol/L	49.40 (33.90, 67.10)	43.40 (31.40, 59.90)	53.50 (37.30, 67.90)	53.10 (35.80, 75.10)	0.0009
EDA, μmol/L	20.90 (17.00, 25.70)	18.20 (15.00, 22.10)	21.70 (18.20, 25.80)	24.20 (19.50, 28.90)	<0.0001
HGLA, μmol/L	151.00 (120.00, 188.00)	131.00 (107.00, 166.00)	156.00 (129.00, 191.00)	166.00 (134.00, 212.00)	0.0006
AA, μmol/L	789.00 (650.00, 928.00)	670.00 (560.00, 794.00)	849.00 (741.00, 951.00)	876.00 (724.00, 1050.00)	<0.0001
C-reactive protein, mg/dL	0.20 (0.08, 0.46)	0.20 (0.07, 0.40)	0.20 (0.09, 0.46)	0.22 (0.09, 0.53)	0.8337

Regarding continuous data, the results are displayed as survey-weighted median (Q1, Q3), with *P*-values determined through survey-weighted linear regression. For discrete data, the findings are given as survey-weighted percentages (95% CI), with *P*-values determined through survey-weighted Chi-square tests. ALA, α-linolenic acid; SDA, stearidonic acid; EPA, eicosapentaenoic acid; DHA, docosahexaenoic acid; LA, linoleic acid; GLA, γ-linolenic acid; EDA, eicosadienoic acid; HGLA, dihomo-γ-linolenic acid; AA, arachidonic acid; DPA, docosapentaenoic acid.

**TABLE 2:** Prevalence of pain in male and female participants at three DHA levels.

Outcomes	All participants (*n* = 1,677)	Lower DHA (*n* = 550)	Middle DHA (*n* = 564)	High DHA (*n* = 563)	*P*-value
Chronic pain in males, n (%)					0.658
Yes	116 (13.70)	48 (15.05%)	36 (12.59%)	32 (13.22%)	
No	731 (86.30)	271 (84.95%)	250 (87.41%)	210 (86.78%)	
Chronic pain in females, n (%)					0.166
Yes	160 (19.28)	54 (23.38%)	51 (18.35%)	55 (17.13%)	
No	670 (80.72)	177 (76.62%)	227 (81.65%)	266 (82.87%)	

### Unadjusted association between baseline variables and chronic pain prevalence

Supplementary Figure 1 illustrates the unadjusted relationships between baseline variables and the prevalence of chronic pain, employing weighted univariate logistic models. The findings revealed that chronic pain prevalence was notably greater in females than in males, with an OR of 1.60 (95% CI, 1.13–2.25; *P* = 0.0185). Chronic pain prevalence increases with age, with an OR of 1.01 (95% CI, 1.00–1.02), *P* = 0.0237. With an increase in the PIR, the occurrence of chronic pain declines, reflected by an OR of 0.85 (95% CI: 0.77–0.94; *P* = 0.0064). Individuals with diabetes showed a notably greater occurrence of chronic pain relative to individuals without the condition, resulting in an OR of 2.29(95% CI, 1.12–4.69; *P* = 0.0394). Compared with current smokers, both former smokers and non-smokers had a significantly lower prevalence of chronic pain, with ORs of 0.44 (95% CI, 0.29–0.66), *P* = 0.0018 for former smokers, and 0.37 (95% CI, 0.26–0.52), *P* = 0.0001 for non-smokers.

### Detection of non-linear associations

**FIGURE 2:**
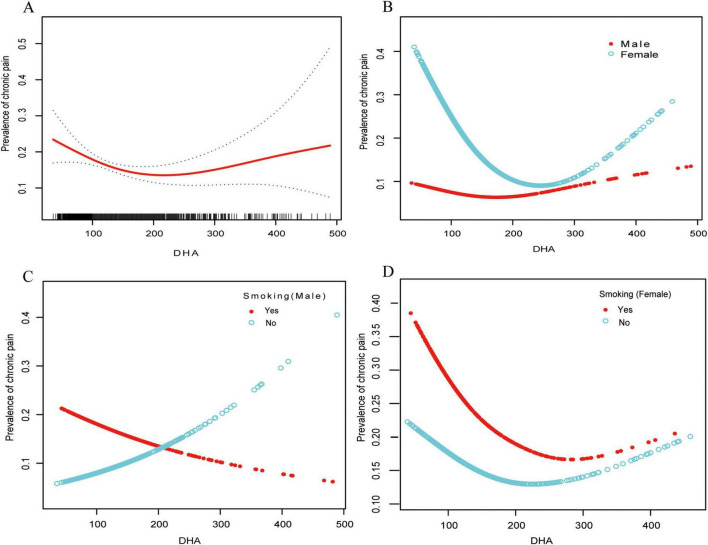
Relationships between serum DHA concentrations and chronic pain prevalence stratified by sex and smoking status. The analysis was conducted using generalized additive models (GAM) to identify non-linear relationships. The solid red line illustrates the smoothed curve modeling the relationship, while the dashed lines indicate 95% confidence intervals. All models were adjusted for age, sex, BMI, race, tobacco use status (never/past/current), alcohol use, education level, Poverty Income Ratio, and diabetes status (yes/no). **(A)** Shows the total study population (*n* = 1,677). **(B)** Shows stratification by gender: females (*n* = 852) and males (*n* = 823). **(C)** Displays the male population stratified by smoking status: male smokers (*n* = 469) and male non-smokers (*n* = 354). **(D)** Displays the female population stratified by smoking status: female smokers (*n* = 373) and female non-smokers (*n* = 479). DHA, docosahexaenoic acid.

We identified a non-linear dose–response association between serum DHA and chronic pain in females (Figure 2 and Table 3). When serum DHA < 187μmol/L, the chronic pain prevalence declined, showing an adjusted OR of 0.92 (95% CI, 0.87–0.97; *P* = 0.019) for every 10-unit rise in serum DHA. When the serum DHA was ≥ 187μmol/L, chronic pain prevalence increased, with an adjusted OR of 1.03 (95% CI, 0.99–1.08; *P* = 0.1578) for every 10-unit rise in serum DHA (Table 3). In males, a liner association was observed between serum DHA and chronic pain (OR = 1.0, 95% CI, 0.97–1.03; *P* = 0.9057).

**TABLE 3:** Threshold effect analysis of baseline DHA on the risk of chronic pain using piecewise linear regression model.

Models	Per-10 unit increase in males (*n* = 847)		Per-10 unit increase in females (*n* = 830)	
	**OR (95%CI)**	***P*-value**	**OR (95%CI)**	***P*-value**
**Model I**				
One line effect	1.00 (0.97, 1.03)	0.9057	0.98 (0.95, 1.01)	0.1219
**Model II**				
Turning point (K)	135		187	
DHA < K	0.94 (0.86, 1.03)	0.1854	0.92 (0.87, 0.97)	0.0019
DHA ≥ K	1.02 (0.98, 1.07)	0.2599	1.03 (0.99, 1.08)	0.1578
*P-*value for LRT test		0.146		0.007

Data were presented as OR (95% CI) *P*-value. Adjusted age, race, education, BMI, smoking status, alcohol use, diabetes, C-reactive protein and Poverty Income Ratio. OR, odds ratio; DHA, docosahexaenoic acid.

Utilizing the generalized additive model, a L-shaped relationship between serum DHA levels and chronic pain prevalence was observed in females. An evaluation of the linear regression model against a two-segment linear regression model resulted in a *P-*value of 0.007 from the log-likelihood ratio test, suggesting that the two-segment model is better suited for fitting the data.

### Interaction between smoking and DHA exposure on chronic pain

In males, a notable relationship between smoking status and DHA exposure was observed (*P*-interaction = 0.0062) (Table 4). Among non-smoking males, higher serum DHA levels were associated with increased odds of chronic pain (OR: 1.06, 95% CI: 1.01–1.11, *P* = 0.0220), with each 10-unit increase in DHA corresponding to a 6% higher odds of reporting chronic pain. In contrast, among male smokers, no significant association between DHA levels and chronic pain prevalence was detected (OR: 0.97, 95% CI: 0.93–1.01, *P* = 0.1463). In females, no significant interaction between smoking status and DHA levels was observed (P-interaction = 0.2830), with neither smoking females (OR: 0.96, 95% CI: 0.92–1.00, *P* = 0.0650) nor non-smoking females (OR: 0.99, 95% CI: 0.95–1.02, *P* = 0.5315) showing a significant association between DHA levels and chronic pain prevalence after adjustment for covariates.

**TABLE 4:** Interaction between smoking and DHA exposure on chronic pain.

Model	Smokers	Non-smokers	*P* interaction
Male			
Crude	0.97 (0.93, 1.01) 0.1083	1.06 (1.01, 1.11) 0.0113	0.0032
Model I	0.96 (0.92, 1.00) 0.0820	1.06 (1.01, 1.10) 0.0172	0.0032
Model II	0.97 (0.93, 1.01) 0.1463	1.06 (1.01, 1.11) 0.0220	0.0062
Female			
Crude	0.97 (0.93, 1.01) 0.1106	1.00 (0.96, 1.03) 0.7904	0.2837
Model I	0.96 (0.92, 1.00) 0.0694	0.99 (0.96, 1.03) 0.6083	0.2644
Model II	0.96 (0.92, 1.00) 0.0650	0.99 (0.95, 1.02) 0.5315	0.2830
Total			
Crude	0.97 (0.94, 1.00) 0.0231	1.02 (0.99, 1.04) 0.2475	0.0130
Model I	0.96 (0.93, 0.99) 0.0122	1.01 (0.98, 1.04) 0.4044	0.0128
Model II	0.96 (0.93, 0.99) 0.0168	1.01 (0.98, 1.04) 0.4904	0.0200

Results in table, OR (95%CI) *P*-value. OR represents the risk of chronic pain prevalence for every 10-unit increase in DHA. Model I, Adjusted for age. Model II, Adjusted for age, race, education, BMI, smoking status, alcohol use, diabetes, C-reactive protein and Poverty Income Ratio.

## Discussion

The research explored the connection between serum DHA concentrations and chronic pain, with an emphasis on the effects of gender and smoking habits. In a cohort of 1,677 individuals with a median age of 46 years, a non-linear, L-shaped relationship was identified in females, indicating that low DHA levels are associated with chronic pain outcomes. In contrast, a linear association was found in males, where non-smokers exhibited a heightened prevalence of chronic pain at elevated DHA levels, while smokers showed no significant associations. These results underscore the intricate relationship between DHA levels, gender, and smoking status concerning chronic pain.

In the context of clinical relevance, it is important to note the significant variation in serum DHA levels across different populations. Our study established tertiles based on the NHANES population distribution: Lower DHA (35.7–106 μmol/L), Middle DHA (107–153 μmol/L), and High DHA (154–489 μmol/L). These values differ substantially from those reported in East Asian populations, where seafood consumption is traditionally higher. For instance, Japanese community studies (14) have reported age-dependent DHA levels of 341.0–353.1 μmol/L in adults aged 40–49 years and 465.8–492.5 μmol/L in those aged 60–69 years, while studies (15) of Chinese pregnant and lactating women have reported ranges of 184.2–445.1 μmol/L. In contrast, pathological states such as ischemic stroke are associated with dramatically lower values (approximately 3.2–3.8 μmol/L) (16). Our observation of a threshold at 187 μmol/L in females, above which the association between DHA and chronic pain becomes non-significant, falls within what would be considered the higher range for Western populations but represents more moderate levels in East Asian contexts. This threshold may indicate that maintaining adequate but not excessive DHA levels relative to population norms could be relevant for pain management in females.

The results partially align with numerous prior empirical studies that have investigated the connection between PUFAs and chronic pain (17–20). One instance is the effectiveness of fish oil intake in easing pain associated with rheumatoid arthritis (21). Furthermore, studies indicate that supplementing with DHA may assist in lowering the initiation and advancement of pain in elderly individuals (22). A Mendelian randomization study further corroborated these results, suggesting that elevated levels of circulating DHA may help relieve abdominal pain (9). These investigations support the observation that elevated DHA levels are associated with a protective benefit against pain. In women, serum DHA concentrations exceeding 187 μmol/L are associated with an increasing trend in chronic pain prevalence. In non-smoking males, there is a positive correlation between DHA levels and chronic pain prevalence. Similarly, a 5-year randomized trial with controlled conditions carried out in the United States discovered that supplementation with omega-3 fatty acids did not enhance joint discomfort related to osteoarthritis (11). In research performed by Hill and colleagues, the outcomes of high-concentration (4.5 g EPA+DHA) compared to low- concentration (0.45 g EPA+DHA) fish oil intake were assessed in individuals suffering from symptomatic knee osteoarthritis over a period of 24 months. The findings indicated that the low-dose group experienced more significant improvements in WOMAC pain and functionality metrics after 2 years (23). This discovery necessitates additional research to determine the reasons behind the greater effectiveness of smaller fish oil doses. The current findings provide some evidence for the potential enhanced efficacy associated with lower amounts of fish oil.

Mechanistically, DHA and various polyunsaturated fatty acids may be associated with reduced inflammation through several pathways. First, DHA can alter the composition and functioning of cell membranes, which in turn affects cellular signaling pathways (24). DHA, a long-chain and unsaturated form of n-3 alpha-linolenic acid (ALA), serves as a key precursor for several unique categories of specialized pro-resolving mediators (SPMs), including D-series resolvins (RvD1-5), maresins, and protectins. These SPMs are potent lipid mediators that actively resolve inflammation rather than simply suppressing it.

In mouse models, DHA and its derived SPMs, such as RvD1, RvD5, and neuroprotectin D1, have demonstrated significant postoperative analgesic effects (25). Furthermore, studies on inflammatory pain models have shown that SPMs derived from DHA can reduce thermal hyperalgesia (26, 27) and mechanical hyperalgesia (28). At the cellular level, these mediators function by modulating ion channels involved in nociception, inhibiting pro-inflammatory cytokine production, and reducing neutrophil infiltration into inflamed tissues.

The anti-inflammatory impacts of DHA can differ from person to person, influenced by factors like gender (29), smoking status (30), and hormone levels (31). Sex hormones likely influence the metabolism of DHA (29), with estrogen potentially enhancing its anti-inflammatory properties (29), which may explain the gender-specific associations observed in our study. Estrogen is known to upregulate the expression of enzymes involved in the conversion of DHA to resolvins and protectins, potentially enhancing their anti-inflammatory effects in women with adequate DHA levels (29). The significant interaction between DHA levels and smoking status in males represents a novel finding in our study. In non-smoking males, elevated DHA levels were associated with higher chronic pain prevalence, which seems counterintuitive given DHA’s anti-inflammatory properties. However, this may be explained by several biological mechanisms: (1) excessive DHA might disrupt the omega-3/omega-6 balance, potentially altering eicosanoid production (32, 33); (2) high levels of DHA could lead to increased lipid peroxidation in certain contexts, generating pro-inflammatory mediators (34); or (3) there may be gene-DHA interactions specific to males that affect pain processing pathways (35).

In contrast, smokers experience greater oxidative stress and chronic low-grade inflammation (36), which might interact differently with DHA metabolism. This implies that in a low-inflammatory context, modest amounts of DHA could provide anti-inflammatory benefits. However, when DHA levels exceed a specific threshold, shifts in biological mechanisms—such as changes in cell membrane fluidity or disruptions in signaling pathways—might result in an altered relationship with pain perception (37).

Beyond these biological mechanisms, several alternative explanations for this interaction warrant consideration. First, dietary patterns often differ substantially between smokers and non-smokers. Smokers typically consume different dietary profiles, with lower intake of fruits, vegetables, and other nutrients that might work synergistically with DHA (38). These different dietary contexts could explain why the same DHA levels might be associated with different pain outcomes in smokers versus non-smokers. Socioeconomic factors may also play a role in this interaction (39). While our models adjusted for education and Poverty Income Ratio, residual socioeconomic confounding might still exist. Socioeconomic factors influence both smoking behavior and dietary access, potentially creating complex relationships that our adjustment strategy may not fully capture. For instance, access to healthcare and pain management resources differs by socioeconomic status and could modify the DHA-pain relationship. The possibility of reverse causality should also be considered—individuals with chronic pain might alter their dietary habits or supplement use in response to pain, potentially in ways that differ between smokers and non-smokers. Non-smoking males with pain might be more likely to increase omega-3 intake as a self-management strategy (40), which could explain the positive association between DHA levels and pain in this group. Conversely, smokers with pain might be less likely to modify their diet, resulting in the different patterns observed. Other lifestyle factors associated with smoking, such as alcohol consumption patterns, physical activity levels, or stress, might modify the relationship between DHA and pain processing. While we adjusted for alcohol consumption, other confounders might remain unaccounted for. For example, smoking is associated with higher stress levels [add reference], which could independently influence both pain perception and inflammatory processes.

This research holds important implications for understanding persistent pain, a prevalent issue that significantly affects public health and imposes a substantial strain on healthcare systems. The results clarify the intricate relationship between serum DHA levels and chronic pain, influenced by factors such as gender and smoking habits. When evaluating DHA levels in relation to pain, it’s crucial to consider a person’s smoking status. For non-smokers, tailored approaches might be needed to maximize potential benefits for chronic pain. In contrast, smokers may require additional research to establish the optimal approach, given the extra complications related to oxidative stress and inflammation caused by smoking.”

It is important to acknowledge that our assessment of chronic pain relied solely on self-reported duration (> 3 months), without clinical confirmation or evaluation of important psychosocial factors such as depression, anxiety, or pain catastrophizing. These psychosocial factors can significantly influence pain perception and chronicity. The absence of more comprehensive pain assessment, including pain intensity, interference with daily activities, or quality-of-life measures, represents a limitation in our study. Additionally, the lack of information on pain management strategies or medication use among participants could confound the observed associations.

### Strengths and limitations

The study presents multiple advantages and limitations that merit consideration. One significant strength is its substantial sample size of 1,677 participants, sourced from the representative NHANES 2003-2004 dataset, which enables a thorough examination of the connection between serum DHA levels and chronic pain. Furthermore, employing advanced statistical techniques, such as generalized additive models, adds to the credibility of the results. Nonetheless, there are important limitations to consider. A primary constraint is the cross-sectional nature of the study, which precludes establishing causal relationships between serum DHA levels and chronic pain. The narrow demographic focus on individuals aged 20 and above within the United States may restrict the applicability of the results to different demographics or age categories. Additionally, it is important to note that our study uses data from 2003 to 2004, approximately two decades ago. During this period, dietary patterns, lifestyle factors, and pain management approaches have evolved significantly. The prevalence of omega-3 supplementation has increased, and public awareness of dietary fats has changed. Therefore, the relationships observed in our study may not fully reflect current associations between DHA and chronic pain in today’s population. Furthermore, the U.S. population sampled in NHANES may not represent dietary patterns and genetic factors in other geographical regions where fish consumption and other sources of omega-3 fatty acids vary considerably. The cross-sectional approach prevents determination of temporal relationships between DHA levels and pain development.

Our definition of chronic pain based solely on duration without accounting for clinical confirmation or psychosocial factors represents a key limitation. Pain is a complex biopsychosocial experience, and our simplified assessment may have led to misclassification in some cases. Future studies should incorporate validated pain questionnaires, clinical evaluations, and assessment of psychological factors to better characterize chronic pain conditions.

To overcome these constraints, upcoming studies should focus on involving larger, more varied populations and utilize longitudinal designs to more clearly clarify the temporal relationships and potential mechanisms involved.

## Conclusion

This research uncovers an intricate, gender-dependent association between serum DHA concentrations and the prevalence of long-lasting pain. In women, the findings indicate a non-linear, L-shaped relationship, implying that both insufficient and excessive DHA levels are associated with different chronic pain outcomes. In men, a direct correlation was observed, revealing that elevated DHA levels were linked to a higher prevalence of chronic pain among non-smokers. These results highlight the significance of considering gender variations and smoking habits when studying DHA levels in relation to chronic pain, suggesting that research approaches should take into account these factors to enhance patient outcomes. From a clinical perspective, these findings indicate that healthcare professionals may want to assess serum DHA levels during chronic pain evaluations, particularly in female patients, as an optimal DHA range might be associated with lower pain prevalence. For male patients, especially non-smokers, tracking DHA levels may aid in recognizing individuals with different risk profiles for chronic pain, facilitating more personalized approaches. Future studies should aim to clarify the mechanisms behind these associations and investigate whether targeted DHA supplementation plans tailored to individual patient profiles could be effective for chronic pain management.

## Data Availability

The raw data supporting the conclusions of this article will be made available by the authors, without undue reservation.
