# Supplementary material for: Gender-specific associations between serum docosahexaenoic acid levels and chronic pain prevalence: a cross-sectional study
Source: Front Med (Lausanne). 2025 May 27;12:1563209. doi: 10.3389/fmed.2025.1563209 (PMC12148845; doi:10.3389/fmed.2025.1563209)
Supplement: Supplementary file 1 [file Data_Sheet_1.doc]

**Gender-Specific Associations Between Serum Docosahexaenoic Acid Levels and Chronic Pain Prevalence: A Cross-Sectional Study**

Supplemental files

**Supplemental Tables.** Covariate Checking and Selection.

**Supplemental figure 1.** Participants inclusion flowchart.

**Supplemental Tables. Covariate Checking and Selection**

VIF Collinearity Screening

|  | Step 1 |
| --- | --- |
| DHA | 1.1 |
| Gender | 1.1 |
| Age | 1.2 |
| Race/ethnicity | 1.2 |
| Education | 1.3 |
| Poverty Income Ratio | 1.3 |
| BMI | 1.1 |
| C-reactive protein | 1 |
| Alcohol | 1.1 |
| Diabetes | 1.1 |
| Smoking status | 1.1 |

1. Examine the relationship between each covariate and the outcome variable (Y) individually.

| Covariates | N | term | beta | Se. | Exp (beta) | 95%CI Low | 95%CI Upp | P.value |
| --- | --- | --- | --- | --- | --- | --- | --- | --- |
| Gender | 1677 | Factor (Gender)2 | 0.4087 | 0.1332 | 1.5049 | 1.1592 | 1.9537 | 0.0021 |
| Age | 1677 | Age | 0.0061 | 0.0035 | 1.0061 | 0.9992 | 1.0130 | 0.0817 |
| Race/ethnicity | 1677 | factor(Race/ethnicity)2 | -0.2375 | 0.1794 | 0.7886 | 0.5548 | 1.1208 | 0.1855 |
|  |  | factor(Race/ethnicity)3 | -0.7706 | 0.1965 | 0.4627 | 0.3148 | 0.6802 | 0.0001 |
|  |  | factor(Race/ethnicity)4 | 0.0665 | 0.3479 | 1.0687 | 0.5404 | 2.1136 | 0.8485 |
|  |  | factor(Race/ethnicity)5 | -1.1852 | 0.6038 | 0.3057 | 0.0936 | 0.9983 | 0.0497 |
| Education | 1677 | factor(Education)2 | 0.0408 | 0.1713 | 1.0417 | 0.7446 | 1.4573 | 0.8116 |
|  |  | factor(Education)3 | -0.3098 | 0.1566 | 0.7336 | 0.5397 | 0.9971 | 0.0479 |
|  |  | factor(Education)7 | -12.0657 | 378.5929 | 0.0000 | 0.0000 | Inf | 0.9746 |
| Poverty Income Ratio | 1677 | Poverty Income Ratio | -0.1103 | 0.0437 | 0.8956 | 0.8221 | 0.9756 | 0.0116 |
| BMI | 1677 | BMI | 0.0200 | 0.0103 | 1.0202 | 0.9999 | 1.0409 | 0.0510 |
| C-reactive protein | 1677 | C-reactive protein | 0.0889 | 0.0504 | 1.0929 | 0.9902 | 1.2063 | 0.0776 |
| Alcohol | 1677 | factor(Alcohol)2 | 0.0660 | 0.1430 | 1.0683 | 0.8072 | 1.4138 | 0.6441 |
| Diabetes | 1677 | factor(Diabetes)1 | 0.5949 | 0.1938 | 1.8128 | 1.2399 | 2.6504 | 0.0021 |
| Smoking status | 1677 | factor(Smoking status)2 | -0.4729 | 0.1341 | 0.6232 | 0.4792 | 0.8105 | 0.0004 |

2. Introduce covariates in the basic model and remove them in the full model to observe changes in the regression coefficient of X
X= DHA

|  |  | Basic model | Complete model |  |
| --- | --- | --- | --- | --- |
| Covariates | +/- term | DHA | DHA | Selected |
|  | Starting regression coefficient | -0.0007 | -0.0013 |  |
| Gender | Factor (Gender) | -0.0011 * | -0.0008 * | Yes |
| Age | Age | -0.0012 * | -0.0012 | Yes |
| Race/ethnicity | Factor (Race/ethnicity) | -0.0009 * | -0.0011 * | Yes |
| Education | Factor (Education) | -0.0005 * | -0.0013 | Yes |
| Poverty Income Ratio | Poverty Income Ratio | -0.0004 * | -0.0015 * | Yes |
| BMI | BMI | -0.0006 * | -0.0013 | Yes |
| C-reactive protein | C-reactive protein | -0.0007 | -0.0013 |  |
| Alcohol | Factor (Alcohol) | -0.0007 | -0.0013 |  |
| Diabetes | Factor (Diabetes) | -0.0009 * | -0.0012 | Yes |
| Smoking status | Factor (Smoking status) | -0.0004 * | -0.0015 * | Yes |

* Indicates a change of more than 10% compared to the initial regression coefficient.

Selected covariates

| Y | X | Selected covariates (Model 1) | Selected covariates (Model 2) |
| --- | --- | --- | --- |
| Chronic pain | DHA | Gender, Age, Race/ethnicity,  Education, Poverty Income Ratio,  BMI, Diabetes, Smoking status | Gender, Age, Race/ethnicity, Education,  Poverty Income Ratio, C-reactive protein,  Diabetes, Smoking status |

Notes:

Criterion 1: Introducing covariates in the basic model or removing covariates in the full model affects the regression coefficient of X by more than 10%.

Criterion 2: Criterion 1 or the covariate's effect on the regression coefficient of Y has a P-value < 0.1.

**Supplemental figure 1.** Forest plot displaying the unadjusted associations between baseline variables and chronic pain prevalence, analyzed using weighted univariate logistic models.


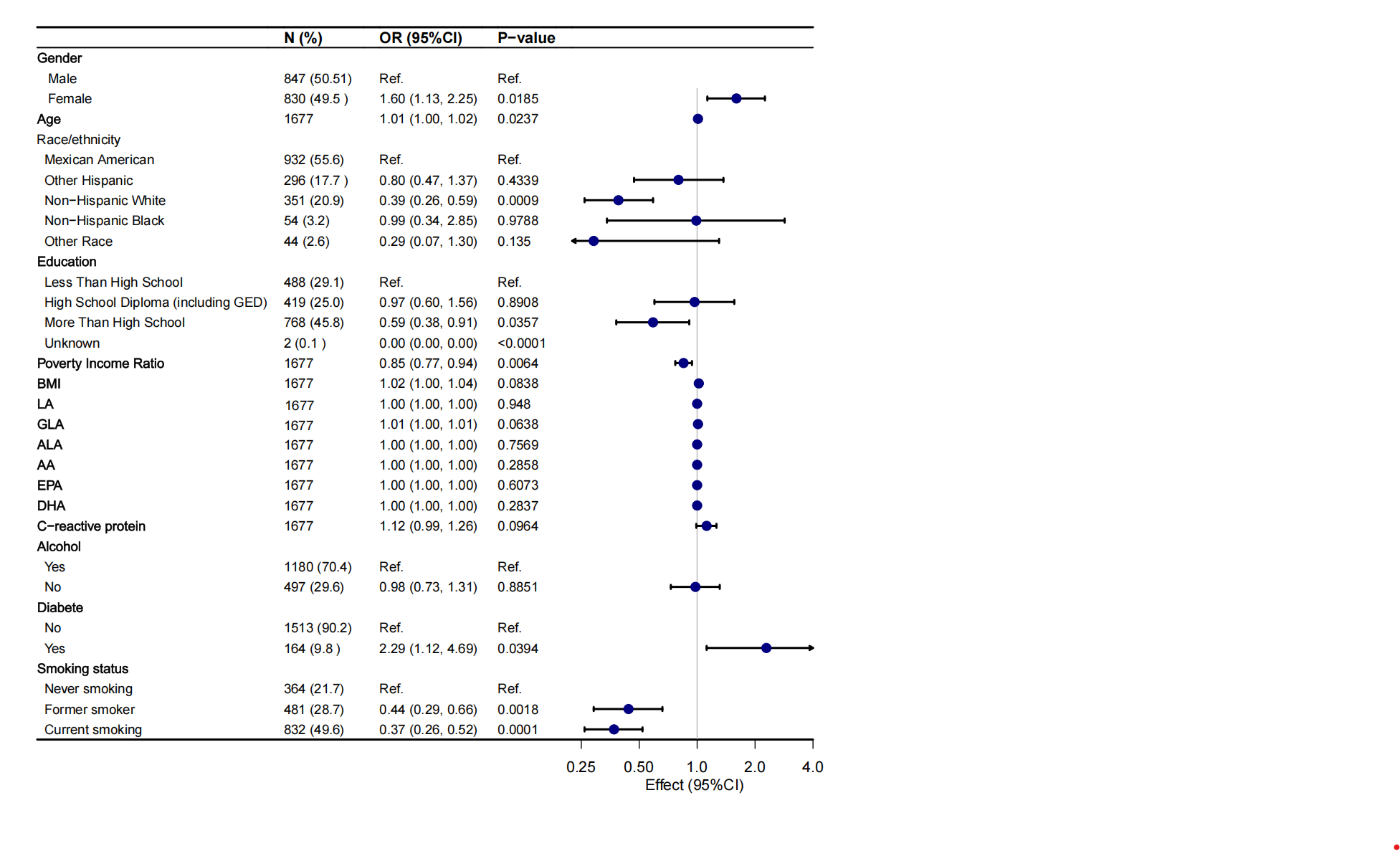


ALA, α-linolenic acid; EPA, eicosapentaenoic acid; DHA, docosahexaenoic acid; LA, linoleic acid; GLA, γ-linolenic acid; AA, arachidonic acid.
